# Hunting and use of terrestrial fauna used by Caiçaras from the Atlantic Forest coast (Brazil)

**DOI:** 10.1186/1746-4269-5-36

**Published:** 2009-11-21

**Authors:** Natalia Hanazaki, Rômulo RN Alves, Alpina Begossi

**Affiliations:** 1 Ecology and Zoology Department, Federal University of Santa Catarina, ECZ-CCB-UFSC, Florianópolis-SC, 88010-970, Brazil; 2 Fisheries and Food Institute, Rua Coronel Quirino 1636 ap 01, Campinas-SP, 13025-002, Brazil; 3 Departamento de Biologia, Universidade Estadual da Paraíba, Av das Baraúnas, 351/Campus Universitário, Bodocongó, 58109-753, Campina Grande-PB, Brazil; 4 Capesca, Preac & CMU (UNICAMP), CP 6023, Campinas-SP, 13083-970, Brazil

## Abstract

**Background:**

The Brazilian Atlantic Forest is considered one of the hotspots for conservation, comprising remnants of rain forest along the eastern Brazilian coast. Its native inhabitants in the Southeastern coast include the Caiçaras (descendants from Amerindians and European colonizers), with a deep knowledge on the natural resources used for their livelihood.

**Methods:**

We studied the use of the terrestrial fauna in three Caiçara communities, through open-ended interviews with 116 native residents. Data were checked through systematic observations and collection of zoological material.

**Results:**

The dependence on the terrestrial fauna by Caiçaras is especially for food and medicine. The main species used are *Didelphis *spp., *Dasyprocta azarae*, *Dasypus novemcinctus*, and small birds (several species of Turdidae). Contrasting with a high dependency on terrestrial fauna resources by native Amazonians, the Caiçaras do not show a constant dependency on these resources. Nevertheless, the occasional hunting of native animals represents a complimentary source of animal protein.

**Conclusion:**

Indigenous or local knowledge on native resources is important in order to promote local development in a sustainable way, and can help to conserve biodiversity, particularly if the resource is sporadically used and not commercially exploited.

## Background

Animals have been used for numerous purposes by human populations for millennia in Brazil [1-6]. The many uses of faunal resources have always stimulated hunting which continues, to a greater or lesser extent, to the present day [1]. Little attention, however, has been given to this use of the biodiversity in Brazil and the few works that have been published on the subject were studies undertaken in the Amazonian region [7-13].

The Brazilian Atlantic forest comprises remnants of rain forest along a narrow strip in the Brazilian coast between latitudes 14° and 21° S. The region of the Brazilian Atlantic forest was the first region occupied by the European colonizers in post-Columbian times in Brazil, and concentrates the most populated cities in this country, such as São Paulo and Rio de Janeiro. In spite of this, native inhabitants of Atlantic forest include both Amerindians and non-Amerindian peoples. This latter group includes the Caiçaras: people of mixed origin, descendants from both Amerindian and European colonizers, also with influences of other cultures such as from African slaves and Japanese immigrants [14].

The livelihood of Caiçaras includes artisanal fishery, small-scale agriculture, and the use of plants for several purposes [15-21]. At some communities they also work in the growing activities associated with tourism. Several authors documented the Caiçara knowledge on the Atlantic Forest, including their knowledge regarding the coastal and marine environment [15,22,23] and their knowledge regarding the terrestrial environment [24-26]. However, the use of terrestrial environments by Caiçaras reveals their connections with plant resources, but seldom with faunal resources. For example, in the Amazonian Rain Forest hunting is an important component of native subsistence strategies, and it also represents a serious threat to biodiversity in some areas [27,28]. On the other hand, there are no conclusive studies about the possible effects of hunting practices among rain forest Caiçaras [29].

In this study our objective is to analyse the use of terrestrial fauna by Caiçaras from the Brazilian Atlantic Forest. We intend to make a brief descriptive analysis about the hunting activities associated with the use of faunal resources in three Caiçara communities from the southern coast of São Paulo State, Brazil. Our underlying hypothesis is that Caiçara communities historically depended from the natural resources for their direct subsistence, and because they inhabit forest areas in the coast these resources comprises both the marine environments and the terrestrial environments.

## Methods

Fieldwork was carried out by one of the authors (NH), as part of her PhD research project, between January 1998 and February 2000, totalising about 80 days of fieldwork along different seasons. We studied the local use of terrestrial fauna in three Caiçara communities located on the South-eastern Atlantic Forest coast (Brazil), named Icapara, Pedrinhas, and São Paulo Bagre (between 24° 40'S - 25° 10'S and 47° 20'-48° 05'W). These settlements are located in the lagoon-estuarine region of Cananéia-Iguape [30].

Icapara had an estimated population of 1,600-2,000 people, living in 350-400 houses. Most of them (65%) were native Caiçaras. The native population of Pedrinhas was of 252 people and about 60 families. At São Paulo Bagre we found 17 families and 78 native residents [19]. Their main economic activities are related to small-scale fisheries, small-scale subsistence farming, extraction of non-timber forest products and tourism-related activities. About 35% of the houses at Icapara and 62% at Pedrinhas were not included in our sample because they belong to recent inhabitants or tourists. The tourism-related activities are the work as housekeepers, renting houses for tourists or, in the case of São Paulo Bagre, fishing for living baits for recreational fisheries or guiding recreational fishers.

The first part of the fieldwork included systematic open-ended interviews including socio-economic and ecological information, such as fishing, agriculture, use of terrestrial animals, and dietary habits [31,32]. The interviews were done with native residents (only adults) and with those living in the region for more than 2 years. We did systematic sampling of the households at Icapara and Pedrinhas, conducting interviews in one in each three houses at Icapara and one in each two houses in Pedrinhas. At São Paulo Bagre we did interviews in all households. After informed consent, in each household we tried to separately interview the main couple, but sometimes one of the residents was absent. A total of 116 persons were interviewed, both men (36%) and women (74%). Data from interviews were checked through systematic observations on the use of local resources, and by collecting zoological material.

Zoological material was identified through photographs and through reports of interviewees correlating photographs and pictures with vernacular names, based on literature [33,34]. Dr. E.Z.F. Setz (Zoology Department, Campinas University) supervised mammal identification. Bird identification was based on bibliography [35-37], and revised by Dr. W.R. Silva (Zoology Department, Campinas University). The species of Mollusca used as medicinal resource was identified by Dr. C. Magalhães (Zoology Department, Campinas University), based on photograph. Collected ants were identified by R. G. Raimundo (Natural History Museum, Campinas University), and were deposited on the Natural History Museum of Campinas University.

Data analysis was done through descriptive statistics and chi-square tests for independence for comparisons among communities, with 5% of significance. We used the frequency of answers for the comparisons between communities.

## Results

### Use of domestic animals as food resources

We observed two main uses of terrestrial fauna among Caiçaras. The first one was as a food resource, and the second one as a medicinal resource (Table 1). Terrestrial animals used as food resource include both native fauna and domestic animals, such as chickens and other fowl species.

**Table 1:** Animals cited in the interviews

Group	Species	Local name	Uses
**Mollusca**			
Bulimulidae	*Megalobulimus *sp.	Caramujo	M

**Birds**			
Cracidae	*Penelope *sp.	Jacu	M
Psittacidae	*Amazona brasiliensis*	Papagaio	O
Turdidae	*Turdus albicolis*	Sabiá-branco	F
Turdidae	*Turdus rufiventris*	Sabiá-laranjeira	F
Turdidae	*Platycichla flavipes*	Sabiá-preto	F
Turdidae	*Turdus amaurochalinus*	Sabiá-pardo	F

**Mammals**			
Agoutidae	*Agouti paca*	Paca	F
Cervidae	*Mazama Americana, M. nana*	Veado, cabrito	F, M
Dasipodidae	*Dasypus novemcinctus*	Tatu	F
Dasyproctidae	*Dasyprocta azarae*	Cutia	F
Didelphidae	*Didelphis aurita*	Guaxica, raposa	F
Felidae	*Leopardus pardalis*	Jaguatirica, onça	M
Hydrochaeridae	*Hydrochaeris hydrochaeris*	Capivara	M, F
Myrmecophagidae	*Tamandua tetradactyla*	Tamaduá	F
Tayassuidae	*Pecari tajacu*	Tateto	F, M

**Reptiles**			
Crocodilidae	*Caiman latirostris*	Jacaré	M, F
Teiidae	*Tupinambis *sp.	Lagarto	M

Non-domesticated terrestrial animals with reports of known uses in 116 interviews with Caiçaras from Pedrinhas, Icapara, and São Paulo Bagre, Brazil. Uses: F: food; M: medicinal; O: ornamental

Chicken raising is more intense at São Paulo Bagre and less intense at Icapara. The proportion of families at the studied communities who raise chicken and fowls are statistically different (χ^2 ^= 16.90, 2 degrees of freedom among the three communities; χ^2 ^= 7.16, 1 d.f. between Icapara and Pedrinhas; χ^2 ^= 16.57, 1 d.f. between Icapara and São Paulo Bagre; χ^2 ^= 10.07, 1 d.f. between Pedrinhas and São Paulo Bagre). This result reflects the different degrees of urbanization of the studied communities. Icapara is the most urbanized community, with smaller backyards. Pedrinhas shows an intermediate situation, being an urbanized area but with larger backyards. Different from these two communities, at São Paulo Bagre the houses had no fences defining their yards.

### Use of native animals as food resources

The use of native terrestrial fauna as a food resource is quite sporadic and it seems to be less intense the in the past. The intensity of this activity decreased after the opening of roads in the region, and after the use of motorized boats on fisheries, and certainly after the environmental legislation [29]. Nearby the region of Cardoso island, at Cananéia municipality, the local inhabitants practiced game activities once a week, in the past [38]. The growth of commercial fisheries as an important activity for the local inhabitants modified this scenario [38]. In the first decade of the 20^th ^century, fishing activities were directed to an incipient market, and by the decade of 1960 fisheries became the most important source of income in all over this coastal region [39]. Other activities such as agriculture and game hunting were gradually abandoned since then.

Regarding to the Brazilian environmental legislation, until 1998 the hunting of native animals was a strictly forbidden activity. After 1998, the new regulations consider that hunting activities are allowed when done to satiate the hunger of the hunter or of his/her family, under circumstances of necessity. In spite of this, hunting of native animals still happens in Atlantic Forest with different intensities, and in some cases is a complementary source of animal protein for Caiçara families. The persistence of hunting activities in Brazil in spite of the well-known illegality of this practice is closely associated with cultural questions and with the fact that in some Brazilian regions these animals can have great nutritional importance to low-income families that cannot obtain sufficient protein resources from domestic animals [1].

Hunting was a sporadic activity until the decade of 1950 among small farmers from the countryside of São Paulo State, and since then it decreased [40]. This activity was related, in some areas, to the defence of cultivated fields, often attacked by small mammals. For other populations of Caiçaras, such as Búzios Island, during the windy days in the winter, birds were the only available protein for islanders [41,42]. This relationship between hunting activities and the defence of cultivated fields probably occurred as well among the coastal communities from the southern part of São Paulo State. Reports from agriculturists indicate that the cultivated fields were frequently attacked by "cabritos" (deer, *Mazama *spp.) and "tatetos" (*Pecari tajacu*), especially on the areas away from the houses. In this context, the same animal species can represent either a potential resource or a potential economic loss or health risk. *Mazama *spp and *Pecari tajacu*, for example, can cause damages to cultivated fields and are used as a food resource. A similar situation was described in the semi-arid region of northeastern Brazil [1], where in contrast to any utilitarian value, some species are hunted because they are perceived to represent risks to human health or to domestic stock (e.g. venomous snakes: *Crotalus durissus*, *Micrurus *sp., *Bothrops *sp.) or cause damage to planted areas (e.g. granivorous birds and rodents) or prey on domestic animals such as the felines. These observations are in agreement with Marques [43], who pointed out that the link between humans and animals is fraught with contradictions and ambiguities, as the native fauna can represent either a resource or a risk to the local people.

**Figure 1:**
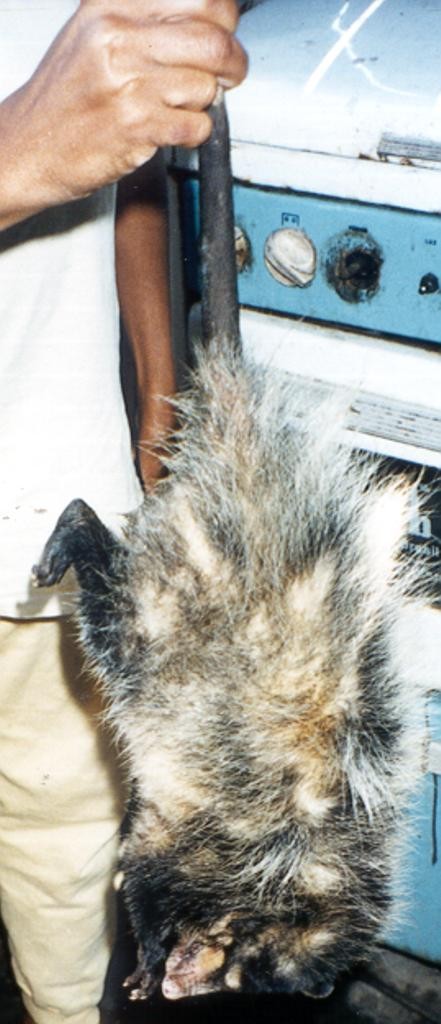
**"Raposa guaxica"**. Opossum, *Didelphis aurita*.

**Figure 2:**
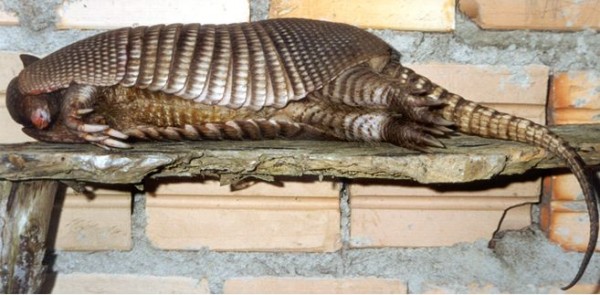
**"Tatu"**. Armadillo, *Dasypus novemcinctus*

**Figure 3:**
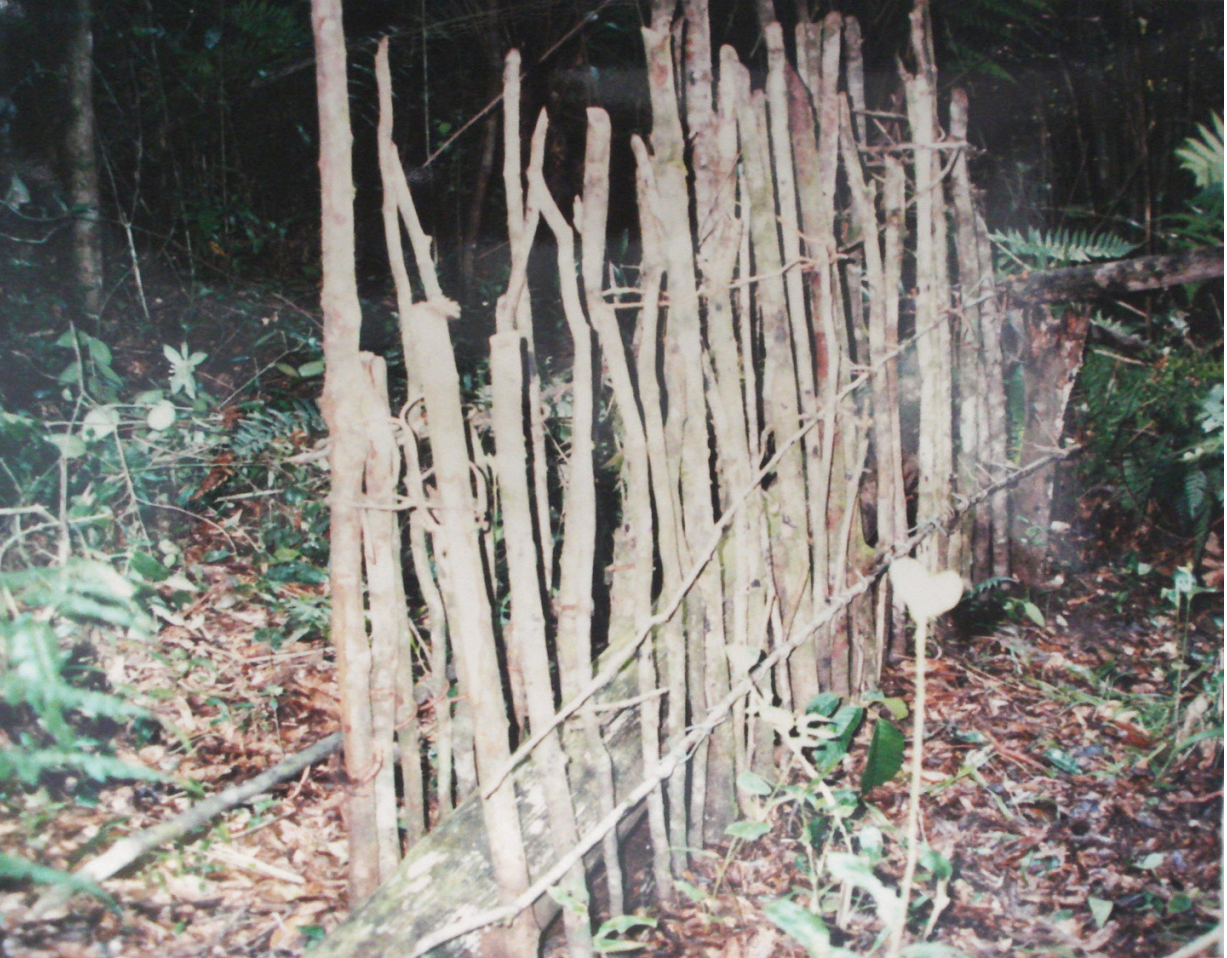
**"Mundéu"**. The artisanal trap called "mundéu", disarmed. The large log in the middle of the trap is expected to fall over the prey.

We observed the occasional hunting of small mammals such as "raposa guaxica" (opossum, *Didelphis aurita*, Figure 1), "cutia" (agouti, *Dasyprocta azarae*), and "tatu" (armadillo, *Dasypus novemcinctus*, Figure 2). Opossum and agouti were hunted with an artisanal trap called "mundéu" (Figure 3), which consists of two parallel rows of sticks fixed on the ground in an "U" shape, and a heavy trunk assembled to fall over the animal when it reaches the bait on the bottom of the "U". Armadillos were hunted with ragged fish nets, with the help of dogs.

Following the foraging behaviour observed for other resources, the hunting of these animals by the Caiçaras does not occur in an indiscriminate way. Hunters are a few individuals in the communities, usually male. Opossum and agouti were hunted during the months before winter (April and May), until the end of June, when the "mundéu" is disassembled. According to the local inhabitants, since the month of June the animals are breeding, with the exception of "tamanduá" (anteater, *Tamandua tetradactyla*). This trend was also observed in ecological studies: in a mark-and-release population study in the Juréia region, also in the Southern part of São Paulo State coast) [44], the marsupials reproduced in a seasonal pattern, resulting in peaks on the population densities on the wet months of December to March, with the entrance of youngest individuals into the population. Marsupials also reproduce between July and September [44]. Caiçaras from the nearby areas of Cardoso Island [38] and Juréia [45] often suspend the hunting activities during periods of reproduction and pregnancy of the animals.

Less frequent than in the past, the hunting of "sabiás" (many species from Turdidae family: *Turdus albicolis, T. Rufiventris, Platycichla flavipes, T. amaurochalinus*) was also observed, especially between May and July. "Sabiás" (thrushes) were caught with "buízas" or "arapucas", which are small traps made from wooden sticks shaped like a pyramid. The traps are assembled near the houses, and avocado (*Persea americana *Mill.) is used as bait. Local inhabitants refer to other hunting techniques used on the past, such as the use of fishing nets along the Turdidae tracks on the ground. This technique allowed greater catches of thrushes with a little effort. The hunting of thrushes was a widespread activity on this region, not only for subsistence but also for commercial purposes [46,47]. Birds were also hunted between May and July at Caiçara communities from the northern coast of São Paulo, such as Búzios Island [41,42]. At this island, a few men used traps and shotguns for birds, while the children used the "bodoque", a double corded bow. Fruits of *Schinus terebinthifolius *Raddi, locally called "aroeira" are put into traps in order to attract the birds. The birds commonly caught at Búzios Island were *Rhamphocelus bresilus *(saddle tanager or "tiê-sangue") and the "sabiás" (thrushes) *Platicychla flavipes*, *Turdus rufiventris*, and *Turdus *spp. In May and June 1987 the Caiçaras from Búzios Island hunted 130 birds to eat, usually mixed with beans [41,42]. It is worth to notice that until now, the impact of hunting activities is certainly greater when shaped by economic forces, such as when the animals are hunted to be sold. For example, some species, such as armadillos, are hunted to attend the demands of urban centres such as Cananéia and Iguape.

**Figure 4:**
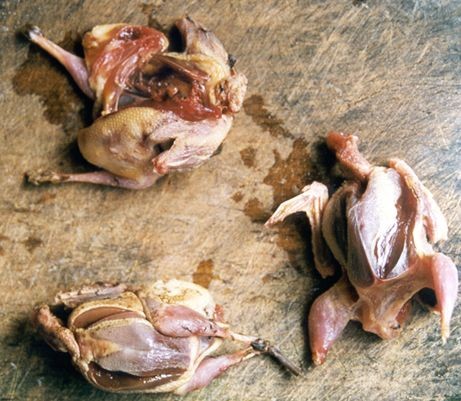
**"Sabiás"**. Three thrushes being prepared to be cooked.

A rough estimative made in one of the studied communities during the days of higher intensities of hunting practices resulted in the maximum values of 1.50 g/day per capita for small birds hunted, and less than 25 g/day per capita for mammals hunted (Figure 4). For some local families, these low values often are the only animal protein available and the use of hunted animals as a food resource was observed in the families with the lowest incomes within the communities. Despite of an observed trend toward the over ingestion of protein due to fishing activities in Caiçara communities [31,48], this is just an average trend, because the distribution of animal protein among families is highly variable. There are some families who do not have easy access to fishing resources. Even among fishermen families, the implicit uncertainty of fisheries results in some fishing trips with no fish caught at all.

Hunting practices aiming terrestrial animals for food is a seasonal activity, and it can be related to the shortage periods of other subsistence activities such fisheries. It is often restricted to a few families of each community, or even to families with less access to fish resources.

### Uses of terrestrial fauna: Animals as medicine

During the interviews, animals hunted in the past were also mentioned, such as the anteater, the "jacu" (*Penelope *sp.), "jacaré" (*Caiman latirostris*), "capivara" (capybara, *Hydrochaeris hydrochaeris*), "tateto", and "veado". Some of these animals were used as a medicinal resource. According to the interviews, 51% of the local inhabitants' knew or use animals for medicine, and 49% did not mention these practices. The frequency of these categories of answers were the same in the three communities (χ^2 ^= 4.29, 2 d.f.) and when pairs of communities were compared (χ^2 ^= 1.52, 1 d.f. for Icapara and Pedrinhas; χ^2 ^= 2.80, 1 d.f. for Icapara and São Paulo Bagre; χ^2 ^= 4.25, 1 d.f. for Pedrinhas and São Paulo Bagre with Yates correction).

The part of the animals mostly used as a medicinal resource is the fat of animals such as "jacaré", "lagarto" (*Tupinambis *sp.), "onça" (*Leopardus pardalis*) and "capivara". Animal fat is used to treat respiratory disorders, designed as bronchitis in a generic way, rheumatism, and earaches. On the first two cases the fat is rubbed over the skin of the sick person. For earaches, the warmed fat is used topically.

Examples of other animal resources used for medicine include the "caramujo-do-mato" (*Megalobulimus *sp.), found in the anthropogenic areas nearby the communities. This mollusk is toasted and triturated, and used to "depurate the blood" and for an unidentified disease called "mores". This species was also medicinal at Búzios Island, its shell being toasted, triturated and applied on wounds [49]. The ox horn toasted with mint (*Mentha *sp.) is used against parasitic worms. The toasted "alecrim" (excrements) of dogs, mixed with "sabugueiro" (*Sambucus asutralis *Cham. & Schltd.), is sometimes used to treat measles.

The use of fatty substances for medicinal purposes is probably of European origin. According to Araujo [50], these substances were widely used on Portugal pharmacopoeia during the 17^th ^and 18^th ^centuries. The use of medicinal animals is mentioned by several authors [2,3,6,7,51], among others], and probably is a common practice for all the human populations [52].

In Brazil, since the 1980s various publications have shown the importance of zootherapy for traditional communities from distinct socio-cultural-environmental landscapes. All the medicinal species mentioned by the interviewees had been recorded in previous studies in Brazil [6,7,53], and in many cases there was full agreement regarding their medicinal uses, which suggests that their use is widespread in the country. Among the Caiçaras there is a slight threshold between what is food and what is a drug: animals may be used as medicine, but are also eaten exclusively in case of illness [54].

### Local knowledge, conservation, and development

The use of natural resources by Caiçaras includes a richness of more than 300 species, including plants, fishes, terrestrial and aquatic animals [20]. The use of different resources varies in intensity and frequency: while medicinal plants have a sporadic use, fishing resources are used daily. Compared with these resources, the terrestrial fauna use is sporadic and seasonal, occurring mainly during the winter months, the season unsuitable for fisheries. Contrasting with a high dependency on terrestrial faunal resources by native Amazonians, the Caiçaras show a high dependency of aquatic faunal resources [15], but a secondary and supplemental dependency on terrestrial faunal resources. Nevertheless, the hunting of animals occasionally represents a complimentary source of animal protein. In the semi-arid region of northeastern Brazil, the practice of hunting is quite common, and the people use animal resources in various ways (for medicinal and ornamental purposes, and as food sources or as pets) - which demonstrates the economic and cultural significance of local fauna to people in this region [1].

The diverse resources used result from practices of combined livelihood activities, which are interrelated along the year. Historically the agriculture and the seasonality of some fish species shaped the livelihood calendar of Caiçaras [15,39,45], and the same was observed in the studied communities. By the month of June new agricultural fields were established, with the fallows and burnings. New fields were planted by August and agricultural activities were alternated with cycles of fish season, such as the "tainha" (mullet, *Mugil platanus*) season during the winter, and the "pescadas" (weakfishes, *Cynoscion *spp., *Macrodon ancylodon*) and "robalos" (snook, *Centropomus *spp.) during the summer [39]. Terrestrial animals were hunted between May and June, during the winter period, when mullet was the only fish abundant. Birds were hunted in the winter, a season that brings windy days in to the coast and fishing became difficult. Local calendars were also related to religious and natural markers, acting as parameters for the livelihood activities. For example, Saint Peter's day (June 29) indicates the end of the mullet season; Saint Paul's day (January 25) marks the beginning of the shrimp (*Litopenaeus schimitii*) season; Saint Thomas' day (December 21) marks the ideal day to plant a banana (*Musa acuminate *Colla) variety called "banana-são-tomé". Natural markers were also observed, such as the singing of certain birds in August reminding the time to "sharpen the hoe and begin to plant". Some natural markers are still present in the Caiçara perpection, such as the groups of "guajú" ants (*Eciton *sp.) on the backyards indicting rain, and the month of May, considered the month of "sabiá", or the month when these birds run in groups, being easier to catch.

These are only a few examples of the local knowledge incorporated into a complex body of knowledge locally constructed. Threatens to the local ecological knowledge are directly related to threatens to the social conditions of production of such knowledge, due to changes on the Caiçara livelihood. Local indigenous knowledge on native resources is important to promote local development in a sustainable way [55], and help to conserve biodiversity, particularly if the resource is used sporadically and when it is not commercially exploited. As discussed elsewhere [27,28], the great threaten to terrestrial faunal resources in tropical forests is habitat destruction. However, a great pressure on terrestrial fauna also occurs due to the demands of urban people living near forested areas.

Hunting practices are usually viewed as high impact activities and an illegal activity within the institutional framework of many countries. Following this understanding, local populations have been considered as important agents causing pressures towards native fauna. Our argument is towards a broader and inclusive point of view regarding hunting activities, where local people can have effective contributions to the conservation of faunal resources, since they depend on these resources for food and medicine.

As stressed by Redford [56], native inhabitants should not be considered as *ecologically noble savages*, despite their deep knowledge about the environment. The great challenge now is find the ways to cope the recent changes affecting these local people and changing their livelihoods and their values, with factors such as the maintenance of the social conditions of production of this local ecological knowledge and the improvement of their life quality. In this sense, attention is needed to include local and indigenous people in conservation and development policies, both at a national and at an international scenario.

## Competing interests

The authors declare that they have no competing interests.

## Authors' contributions

NH: Ethnozoological data and analysis of taxonomic aspects. Authors: writing of the manuscript, literature survey and interpretation. All authors read and approved the final manuscript.
